# Genomic instability influences the transcriptome and proteome in endometrial cancer subtypes

**DOI:** 10.1186/1476-4598-10-132

**Published:** 2011-10-31

**Authors:** Jens K Habermann, Nana K Bündgen, Timo Gemoll, Sampsa Hautaniemi, Caroline Lundgren, Danny Wangsa, Jana Doering, Hans-Peter Bruch, Britta Nordstroem, Uwe J Roblick, Hans Jörnvall, Gert Auer, Thomas Ried

**Affiliations:** 1 Laboratory for Surgical Research, Department of Surgery, University of Lübeck, Germany; 2 Computational Systems Biology Laboratory, Biomedicum Helsinki and Institute of Biomedicine, University of Helsinki, Finland; 3 Department of Gynaecological Oncology, Cancer Center Karolinska (CCK), Karolinska University Hospital Solna, Sweden; 4 Department of Medical Biochemistry and Biophysics, Karolinska Institutet, Stockholm, Sweden; 5 Unit of Cancer Proteomics, Biomics Center Karolinska, Karolinska Institutet, Stockholm, Sweden; 6 Genetics Branch, National Cancer Institute, NIH, Bethesda, MD, USA

**Keywords:** aneuploidy, endometrial carcinoma, genomic instability, comparative genomic hybridization, expression arrays, pathway analysis, UPSC

## Abstract

**Background:**

In addition to clinical characteristics, DNA aneuploidy has been identified as a prognostic factor in epithelial malignancies in general and in endometrial cancers in particular. We mapped ploidy-associated chromosomal aberrations and identified corresponding gene and protein expression changes in endometrial cancers of different prognostic subgroups.

**Methods:**

DNA image cytometry classified 25 endometrioid cancers to be either diploid (n = 16) or aneuploid (n = 9), and all uterine papillary serous cancers (UPSC) to be aneuploid (n = 8). All samples were subjected to comparative genomic hybridization and gene expression profiling. Identified genes were subjected to Ingenuity pathway analysis (IPA) and were correlated to protein expression changes.

**Results:**

Comparative genomic hybridization revealed ploidy-associated specific, recurrent genomic imbalances. Gene expression analysis identified 54 genes between diploid and aneuploid endometrioid carcinomas, 39 genes between aneuploid endometrioid cancer and UPSC, and 76 genes between diploid endometrioid and aneuploid UPSC to be differentially expressed. Protein profiling identified AKR7A2 and ANXA2 to show translational alterations consistent with the transcriptional changes. The majority of differentially expressed genes and proteins belonged to identical molecular functions, foremost *Cancer, Cell Death*, and *Cellular Assembly and Organization*.

**Conclusions:**

We conclude that the grade of genomic instability rather than the histopathological subtype correlates with specific gene and protein expression changes. The identified genes and proteins might be useful as molecular targets for improved diagnostic and therapeutic intervention and merit prospective validation.

## Background

Endometrial cancer is the most common malignancy of the female genital tract in the Western world and the fourth common cancer in women [1]. In general it is considered to have a favorable prognosis since it usually becomes symptomatic at an early tumor stage. Thus, about 70% of the affected women are detected at tumor stage I. At this stage, the mean survival of five years has been estimated to be 87%. However, one histopathological subtype, uterine papillary serous cancer (UPSC), presents with an aggressive clinical course characterized by early metastasis, reduced survival rates and inferior prognosis compared to endometrioid carcinomas [2]. Next to histopathology, tumor stage and tumor grade are known to be the most influencing prognostic factors [3].

In breast, prostate and colorectal cancer, also DNA aneuploidy has been reported to be an independent prognostic marker [4-6]. In endometrial cancer, patients with diploid cell populations have a more favorable 5-year survival rate of 94% as opposed to those with aneuploid malignancies (83%) [7]. Aneuploidy can be assessed at the chromosomal level by comparative genomic hybridization (CGH) [8]. Interestingly, CGH results have shown a conserved pattern of chromosomal gains and losses that is distinct and characteristic for different epithelial malignancies [9]. In carcinomas of the vagina the most frequent aberration detected is a gain of 3q [10], while in endometrial carcinomas, copy number gains were mapped to chromosome arms 1q, 3q, 8q, and 10q [11-13]. The predominance of these tumor entity specific chromosomal alterations leads to increased expression of resident genes that seems to be independent of tissue and/or cell type [14] and gives an irreversible disturbance of transcriptional regulation in aneuploid cells [15].

**Figure 1:**
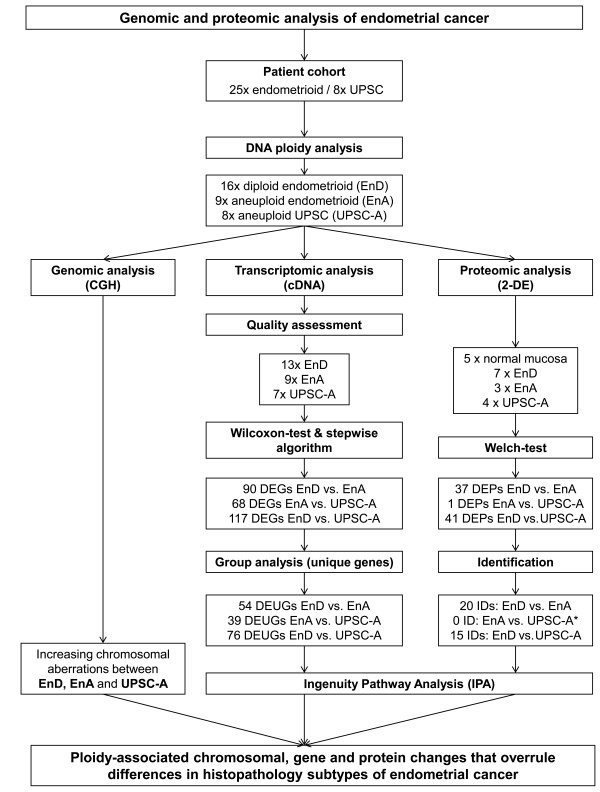
**Technical workflow of the study design**. * No protein was identified in the EnA vs. UPSC-A comparison due to extremely weak abundance of the protein spot in the polyacrylamide gel.

Against this background we now evaluated whether genomic instability correlates with chromosomal alterations and impacts on gene and protein expression changes in endometrial carcinomas. We utilized well-characterized surgical specimens of endometrial cancer representing different histopathological subtypes which are associated with a distinct prognosis (Figure 1).

## Results

Here we describe a comprehensive evaluation of aneuploidy-associated alterations of the genome, transcriptome, and proteome in different histopathological subtypes of endometrial cancer. We were particularly interested in identifying chromosomal alterations that underlay aneuploidy and how these might impact on transcriptional and translational changes and thereby influence patients' prognosis.

### Genomic instability

**Figure 2:**
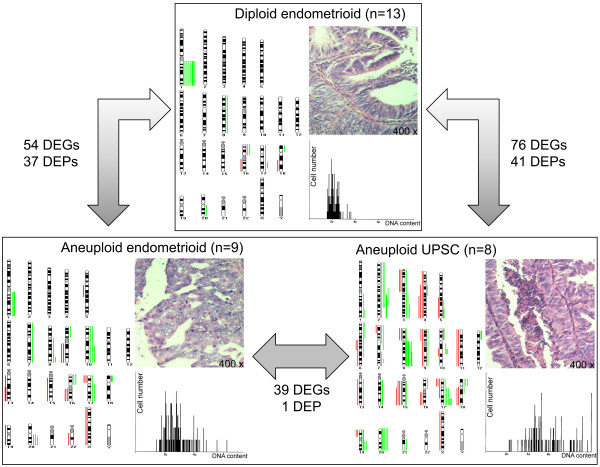
**Examples of ploidy types and number of differential expressed genes (DEGs) and proteins (DEPs)**. DNA histograms show DNA content on the x-axis and the total number of cell on the y-axis.

Of the cancerous samples, 16 of the 25 endometrioid carcinomas showed diploid cell distribution pattern (EnD) and nine presented with aneuploid cell populations (EnA), while all eight UPSC tumors were classified as aneuploid (UPSC-A). Representative histograms for each group are provided in Figure 2. The mean value of the DNA stem line increased from 2.23c in the EnD group to 2.98c in the EnA and 3.06c in the UPSC-A group (p < 0.004).

The "stemline scatter index" (SSI) measures the clonal heterogeneity of the constituent tumor cells and is calculated as the sum of (a) the percentage of cells with DNA content values in the S-phase region (S-phase), (b) the percentage of cells with DNA content values exceeding twice the modal value plus 1c (G2 exceeding rate), and (c) the coefficient of variation (CV) of the respective tumor stemline [16]. In our study, all but one diploid and all aneuploid carcinomas showed SSI values above the threshold of overall genomic instability (of 8.8) found for breast carcinomas [16]. The mean SSI values of 26.9 (EnD), 45.4 (EnA), and 53.5 (UPSC-A) now found indicate increasing genomic instability for aneuploid and UPSC subtypes (p < 0.004). The increasing degree of genomic instability in the EnA and UPSC-A groups compared to the EnD group was also reflected in an increase in chromosomal copy number changes as measured by CGH. A detailed summary and comparison of chromosomal aberrations found is presented in Figure 2. Chromosomal imbalances in the EnD tumors were mostly restricted to gains of chromosome 1q (33%) and 16p (11%). In contrast, EnA tumors showed diverse changes, including a gain of 10q, 20q (both 33%), and 1q, 8q, 10p, 16p and 17q (all 22%). These changes were accompanied by frequent losses of 9q, 16q, 17p, 19p, 19q and 22q (all 22%) (Additional file 1). In the UPSC-A malignancies, chromosomal aberrations affected also chromosomal regions that were not changed in the endometrioid tumors. UPSCs presented in 62% a gain of 2q, 8q, 17q, and 20p and in 50% a gain of 20q. The most frequent loss was observed for 15q (50%).

The increasing number of chromosomal aberrations between the EnD and EnA carcinomas and between the EnA and UPSC-A malignancies was furthermore reflected by the average number of copy number alterations (ANCA), calculated as the sum of all detected chromosomal aberrations and divided by the number of cases analyzed. The ANCA value increased from 0.041 (EnD) to 0.145 (EnA) and up to 0.429 (UPSC-A) (p < 0.001). The same trend was found for the average number of regional amplifications (ANRA, calculated as the sum of all amplifications and divided by the number of cases analyzed). The ANRA amounted to 0.003 in the EnD group, increased to 0.014 in the EnA group and to 0.065 in the UPSC-A group (p < 0.002).

### Gene expression profiling

We applied the Wilcoxon test with permutation test and the stepwise algorithm [17] to identify differentially expressed genes for pair-wise comparisons of the three groups. Considering only those genes that were commonly detected by both approaches, we found that 54 genes were differentially expressed between EnD and EnA samples. A total of 39 differentially expressed genes (DEGs) defined expression differences between the aneuploid malignancies of endometrioid or UPSC histology (EnA and UPSC-A). However, the vast majority-76 genes-was differentially expressed between the groups distinguished by different histology and ploidy status, namely EnD and UPSC-A (Table 1). All genes were unique for pair-wise group comparison. The gene lists describing differences between all groups are listed in Additional file 2.

**Table 1:** Overview of significantly expressed genes

Wicoxon test (p < 0.05)		
EnD versus EnA	EnD versus UPSC-A	EnA versus UPSC-A

478	576	276

**Stepwise Analysis (Up/Down)**		

EnD versus EnA	EnD versus UPSC-A	EnA versus UPSC-A

140/118	195/179	165/154

**Genes present in both analyses**		

EnD versus EnA	EnD versus UPSC-A	EnA versus UPSC-A

90	117	68

**Genes unique for each group comparison**		

EnD versus EnA	EnD versus UPSC-A	EnA versus UPSC-A

54	76	39

EnD, endometrioid diploid

EnA, endometrioid aneuploid

UPSC-A, UPSC aneuploid

When mapping the differentially expressed genes to their chromosomal location, we found that the deregulation of 114 out of 275 (41.45%) genes could be attributed to chromosomal copy number changes.

### Correlation of Gene and Protein expression changes

Mapping differentially expressed proteins (DEPs) previously detected by two-dimensional gel electrophoresis [18] to the chromosomal location of their corresponding genes showed that in the EnD versus EnA comparison 11 of 20 proteins (55%) and in the EnD versus UPSC-A comparison 7 of 15 (47%) mapped to positions that were affected by copy number changes (Additional file 3).

For 5 of the 35 identified proteins, the corresponding cDNA was included on our microarray platform. Two of the corresponding genes, *AKR7A2 *and *ANXA2*, showed a similar trend in transcriptional expression as observed for the translational changes. However, both genes did not reach our significance levels of the gene expression analysis: *AKR7A2 *was down-regulated in EnD versus UPSC-A, while *ANXA2 *showed an up-regulation in EnA versus EnD and in UPSC-A versus EnD.

### Functional annotation of DEGs and DEPs that discern the EnD, EnA, and UPSC-A tumors using Ingenuity Pathway Analysis

Using Ingenuity Pathway Analysis (IPA), differentially expressed genes and proteins that discerned EnD, EnA, and UPSC-A were functionally annotated. An overview of all networks found by transcriptomic and proteomic profiling is provided in Table 2.

**Table 2:** IPA analysis overview

Comparison	Analysis	Top networks	Score	Top Diseases and Disorders	p-Value	# of Molecules	Top Molecular and Cellular Functions	p-Value	# of Molecules
EnD vs. EnA	Transcriptomics	Lipid Metabolism, Small Molecule Biochemistry, Vitamin and Mineral Metabolism	48	Cancer	< 0.0161	23	Lipid Metabolism	< 0.0184	6
		Lipid Metabolism, Small Molecule Biochemistry, Genetic Disorder	30	Hematological Disease	< 0.0132	12	Small Molecule Biochemistry	< 0.0184	10
		Gene Expression, Nutritional Disease, Cellular Development	22	Gastrointestinal Disease	< 0.0105	9	Vitamin and Mineral Metabolism	< 0.0175	12
	
	Proteomics ^&^	Cellular Assembly and Organization, Nucleic Acid Metabolism, Small Molecule Biochemistry	25	Neurological Disease	< 0.0106	7	Cellular Growth and Proliferation	< 0.0382	7
				Genetic Disorder	< 0.0486	8	Cell Morphology	< 0.0297	4
				Cancer	< 0.0394	7	Cellular Assembly and Organization	< 0.0346	6

EnD vs. UPSC-A	Transcriptomics	Organism Injury and Abnormalities, Cardiac Necrosis/Cell Death, Cell Death*	44	Cancer	< 0.0196	31	Cellular Growth and Proliferation	< 0.0212	28
		**Organ Morphology, Reproductive System Development and Function, Skeletal and Muscular Disorders ^#^**	28	Genetic Disorder	< 0.0234	47	Cell-To-Cell Signaling and Interaction	< 0.0234	11
		Cellular Development, Cellular Growth and Proliferation, Cancer	20	Reproductive System Disease	< 0.0196	12	Cell Death	< 0.0234	26
		Cardiovascular Disease, Hematological Disease, Skeletal and Muscular Disorders	19						
		Endocrine System Development and Function, Small Molecule Biochemistry, Gene Expression	15						
	
	Proteomics ^&^	Lipid Metabolism, Small Molecule Biochemistry, Cell Morphology *, ^#^	25	Cancer	< 0.0428	7	Amino Acid Metabolism	< 0.0150	2
				Gastrointestinal Disease	< 0.0214	4	Cell Morphology	< 0.010	1
				Inflammatory Disease	< 0.0479	2	Cellular Assembly and Organization	< 0.0125	2

EnA vs. UPSC-A	Transcriptomics	Cardiovascular System Development and Function, Cell Cycle, Lipid Metabolism	28	Cardiovascular Disease	< 0.0430	12	Gene Expression	< 0.0245	7
		Cell Death, Cellular Movement, Hematological System Development and Function	28	Development Disorder	< 0.0301	5	Cell Death	< 0.0338	15
		Cellular Assembly and Organization, Cellular Function and Maintenance, Cell Signaling	22	Connective Tissue Disorder	< 0.0487	9	Cellular Movement	< 0.0485	8

& Refers to Gemoll et al. [18]

* Overlapping of marked networks via platelet-derived growth factor beta polypeptide (simian sarcoma viral (v-sis) oncogene homolog) dimer (PDGFBB)

# Overlapping of marked networks via beta-Actin (ACTB)

EnD, endometrioid diploid

EnA, endometrioid aneuploid

UPSC-A, UPSC aneuploid

**Figure 3:**
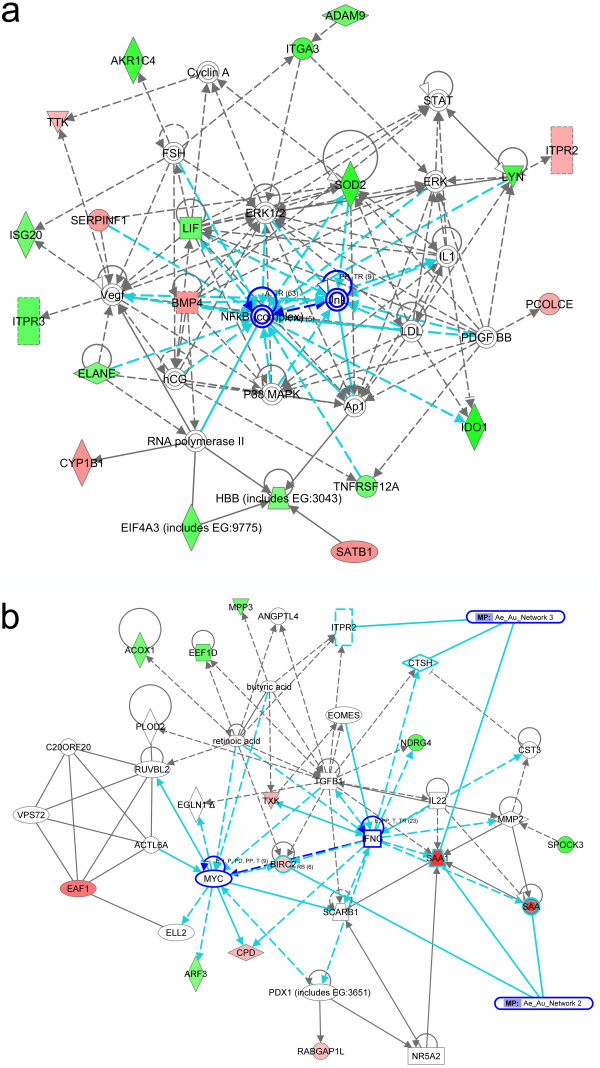
**IPA-based pathway analysis of differential expressed genes**. **(a)** Red and green designations indicate over- and underexpressed genes in the respective groups. Blue arrows and circulations indicate central nodes of the networks. **(b)** Red and green designations indicate over- and underexpressed genes in the respective groups. Blue arrows and circulations indicate central nodes of the networks.

For the comparison of EnD versus EnA, 45 (83%) of the 54 DEGs were recognized in the IPA database and resulted in three networks. The highest ranked network with a score of 48 comprised 20 of the DEGs. These genes interacted in a network (Figure 3a) associated with *Lipid Metabolism, Small Molecule Biochemistry*, and *Vitamin and Mineral Metabolism*. NFkB, Jnk and ERK1/2 were central nodes of this network and associated with diseases and functions regarding *Cancer, Hematological Disease*, and *Gastrointestional Disease *(p < 0.00001 to p < 0.0161). The second highest network (score of 30) comprised 14 of the DEGs and was associated with *Lipid Metabolism, Small Molecule Biochemistry*, and *Genetic Disorder*. The third network (score of 22) comprised 11 of the DEGs and was associated with *Gene Expression, Nutritional Disease *and *Cellular Development*.

The comparison between the aneuploid carcinomas (EnA versus UPSC-A) allowed 33 (84.6%) of all 39 DEGs for IPA analysis. Three overlapping networks reached the level of significance, with a score of 28 to 22. The top network (score of 28) was associated with *Cardiovascular System Development and Function, Cell Cycle, Lipid Metabolism *and particularly with the canonical pathways *Gene Expression *(p < 0.0245) and *Cell Death *(p < 0.0338). This network interacts via BIRC2, SAA, and SAA1 with the second highest network (score of 28), associated with *Cell Death, Cellular Movement*, and *Haematological System Development and Function*, and via CTSH, ITPR2, and SAA1 with the third highest network (score of 22), associated with *Cellular Assembly and Organization, Cellular Function and Maintenance*, and *Cell Signalling*) (Figure 3b). Interestingly, SAA1 connected as well network 2 and network 3 with each other. IFNG, TGFB, MYC, and NFkB act as central nodes in these networks.

In comparisons of EnD versus UPSC-A, a total of 67 (88.2%) of the 76 DEGs were part of the IPA database. Here, we could define five overlapping networks with the first one reaching a score of 44 including 20 of the DEGs being associated with pathways of *Organismal Injury and Abnormalities, Cardiac Necrosis/Cell Death*, and *Cell Death*. Network 2 obtained a score of 28, comprised 14 DEGs and revealed *Organ Morphology, Reproductive System Development and Function*, and *Skeletal and Muscular Disorders *pathways. The third network reached a score of 20, comprised 11 of the DEGs and was associated with *Cellular Development, Cellular Growth and Proliferation*, and *Cancer*. The fourth highest ranked network reached a score of 19, comprised 11 of the DEGs and was associated with *Cardiovascular Disease, Hematological Disease*, and *Skeletal and Muscular Disorders*. The fifth network consists of 9 DEGs and reached a score of 15. Associated network functions were *Endocrine System Development and Function, Small Molecule Biochemistry*, and *Gene Expression*. All networks were associated with *Cancer (p < 0.0196), Genetic Disorder *(p < 0.0234), *Cellular Growth and Proliferation *(p < 0.0212), and *Cell-To-Cell Signalling and Interaction *(p < 0.0234). Three remaining networks obtained one focus gene only and failed the level of significance with a score < 5. Involved genes of all networks are presented in Additional file 4.

A network comparison analysis between all significant networks mentioned above and their corresponding protein profiling networks showed three interacting networks connected via ACTB and PDGFBB (Table 2), thus representing a close relation of transcriptomics and proteomics data.

## Discussion

We mapped ploidy-associated chromosomal aberrations and identified corresponding gene and protein expression changes in endometrial cancers of different prognostic subgroups, including diploid (n = 16) and aneuploid (n = 9) endometrioid, and aneuploid uterine papillary serous cancer (n = 8) malignancies. CGH revealed ploidy-associated specific, recurrent genomic imbalances comprising gains of chromosome arms 1q, 3q, and 8q, as well as losses of 4q and 15q. Since these alterations dominate recurrent pattern of chromosomal imbalances characterizing endometrial malignancies, the genes located on such aberrant chromosome loci might play a key role in initiation and/or progression of endometrial malignancies.

The identified genes with altered expression changes belong to several functional groups. These genes are involved in functions related to fundamental biological processes known to be affected in cancer. For instance, *ATF3, DNMT3B, LMo2*, and *TCF12 *affect DNA binding, transcriptional activation and proliferation. Interestingly, a higher proliferation rate of aneuploid as compared to diploid endometrial carcinomas has been observed [19].

Another group includes genes that code for enzymatic proteins like proteases, reductases, and transferases. Many of these genes, such as aflatoxin B 1 aldehyde reductase member 2 (*AKR7A2*), v-yes-1 Yamaguchi sarcoma viral related oncogene homolog (*LYN*), or cytochrome b reductase 1 (*CYP1B1*) have not been reported in prior studies of endometrial cancer and thus provide novel potential targets for diagnosis and treatment. *AKR7A2 *(located at 1p36.13) is a Golgi-associated AKR7 family member and is expressed in a broad range of tissues [20]. AKR7A2 is involved in the detoxification of aldehydes and ketones in the phase I metabolism. AKR7A2 protein levels are elevated in the cerebral cortex of patients with Alzheimer disease [21]. Our results of the expression analysis indeed proved the proteomic data that demonstrate an AKR7A2 overexpression in EnD compared to UPSC-A [18]. *LYN *(located at 8q13) is among the highly ranked signature genes overexpressed in EnD versus EnA carcinomas and documents a strong correlation between DNA and RNA analysis. LYN is a member of the Src-family kinases, a family of non-receptor tyrosine kinases. LYN was of particular interest because as a kinase, it is "druggable" and might provide a therapeutic opportunity for targeting endometrial cancer. Choi et al. identified LYN as a possible new therapeutic target with particular relevance to clinically aggressive basal-like breast cancer [22].

A further group consists of genes and proteins involved in mechanisms of transport and protein binding. Annexins are characterized by their capacity to bind to phospholipids in the presence of calcium ions and their susceptibility to phosphorylation and dephosphorylation. Belonging to subfamily A of annexins, human annexins are further classified as annexin A1-A11 and A13. One isoform, *ANXA2 *(Annexin A2; located at 15q22.2) was found significantly upregulated as detected in our proteomic approach and proved the same trend with respect to gene expression [18]. ANXA2 is located in the cytoplasm as a monomer (heavy chain of 36 kDa, or p36) or in a complex with a member of the S100A10 [23]. There is evidence of a relationship between proteases and extracellular matrix (ECM) proteins through ANXA2 whereby ANXA2 may facilitate the reorganization of the ECM in physiological and pathological processes such as tumor invasion [24]. Therefore, the over-expression of ANXA2 in colorectal and gastric carcinomas alludes to a correlation with invasiveness and poor prognosis and is in line with our analysis [25,26]. Further, we found *Wnt-7a *(wingless-type MMTV integration site family, member 7A; located at 3p25) to be more expressed in EnA samples compared to EnD cancers. *Wnt *genes are associated with cellular responses such as proliferation, cell fate determination or specification, apoptosis and oncogenesis. The *Wnt-7a *gene is detected at high levels in the female reproductive tract [27]. Observations suggest that disruption of normal Wnt-7a expression by diethylstilbestrol and other estrogenic compounds leads to altered uterine cytoarchitecture and might be a mechanism ultimately causing neoplasia in the reproductive tract [28].

In the comparison of EnD and UPSC-A cases we found that the progesterone receptor (*PGR*; located at 11q22-q23) gene was significantly lower expressed in UPSC-A cases. *PGR *showed the highest ratio of differential expression overall. Furthermore, lower expression in UPSC-A was associated with a loss of the whole chromosome 11 in 25% of the UPSC-A cases whereas in diploid cases no alteration could be detected. PGR encodes a member of the steroid receptor superfamily. The encoded protein mediates the physiological effects of progesterone, which plays a central role in reproductive events associated with the establishment and maintenance of pregnancy. Excessive estrogen stimulation, unbalanced by progesterone, might play a central role in the development of endometrial cancer across all ethnic populations [29,30]. Progesterone ameliorates estrogen-induced proliferation by interacting with its receptor [31,32], primarily through two functionally distinct PGR isoforms. It could be shown that hormone therapy of PGR-positive patients increases their response rate substantially from 8-17% to 37-89% [33]. Our data exhibit that particularly patients with diploid carcinomas could benefit from hormone therapy and that ploidy status assessment could assist in patient stratification.

The comparison between EnA and UPSC-A revealed one network including the differentially expressed genes *BIRC2 *(baculoviral IAP repeat-containing 2; located at 11q22), *BIRC3 *(baculoviral IAP repeat-containing 3; located at 11q22), *MAP3K5 *(mitogen-activated protein kinase kinase kinase 5; located at 6q22.33), and *SAA1 *(serum amyloid A1, located at 11p15.1). All four genes are associated with different cancer types in general but a connection to endometrial cancer has not been described so far. Interestingly, all four genes are lower expressed in the UPSC-A cancers. These findings are in accordance with a loss of the according gene loci on chromosome 6 and 11 in up to 25% of the UPSC-A cases whereas in the EnA cases no alteration could be detected. BIRC2 and BIRC3 are members of a family of proteins that inhibit apoptosis by binding to tumor necrosis factor receptor-associated factors TRAF1 and TRAF2, probably by interfering with activation of ICE-like proteases. These encoded proteins inhibit apoptosis induced by serum deprivation and menadione, a potent inducer of free radicals. Cheng et al. showed that BIRC2 overexpression might play a critical role for mammary carcinogenesis associated with p53 mutations [34]. It is of interest that overexpression of BIRC2 has been recently proposed to be associated with luminal subtype B of breast cancer [35]. MAP3K5 (also known as apoptosis signal-regulating kinase 1; ASK1) has been widely accepted as one of the key components regulating reactive oxygen species (ROS) [36]. There is evidence suggesting that oxidative stress contributes to the pathogenesis of prostate cancer [37]. SAA1, a high-density lipoprotein (HDL)-associated apolipoprotein, is an acute-phase protein which is elevated in response to trauma, inflammation, and neoplasia [38]. SAA1 has several functions such as tumor cell invasion and metastasis by induction of cell adhesion and migration through induction of enzymes degrading the ECM and inhibition of cell attachment to ECM proteins by SAA derived peptides [39-41]. Cumulatively, these properties might place SAA as an ECM-associated adhesion protein, with a potential role in tumor pathogenesis.

## Conclusions

We detected that chromosomal copy number alterations do impact on gene expression changes. In addition, differentially expressed genes and proteins interacted within overlapping networks (Table 2). This was proved further by two differentially expressed proteins, AKR7A2 and ANXA2, showing similar gene expression alterations. Overall, we have identified different and specific expressions patterns between 16 diploid endometrioid-, 9 aneuploid endometrioid-cancers, and 8 aneuploid UPSCs with CGH, RNA microarray and two-dimensional gel electrophoresis. We have revealed a number of aberrantly regulated genes and proteins that are potential biomarkers for an improved diagnosis and prognostication in endometrial cancers.

## Methods

### Patient Samples

Fresh tumor material was collected from women who underwent hysterectomy for endometrial cancer at the Department of Obstetrics and Gynecology at the Karolinska University Hospital, Stockholm, Sweden, during 1997 and 2003. Clinical material was collected from surgically removed tumors adhering to the approval of the local ethical review board. Carcinomas were diagnosed on H&E-stained tissue sections and graded according to the FIGO classification [42]. Patients treated with neoadjuvant therapy were excluded. A total of 25 endometrioid carcinomas and 8 UPSCs were randomly selected for ploidy assessment as well as genomic, and transcriptomic analysis. Proteomic analysis of according samples was reported earlier [18]. Data on ploidy, histopathologic subtype, stage, age, observation time, and survival status are provided in Table 3. After surgery, clinical tissues were first used for touch preparation slides for ploidy assessment and then snap frozen until further processing. Snap frozen specimens were divided into one part for protein expression and one part for DNA and RNA extraction. In addition, paraffin-embedded specimens of the same tumors were used for histopathology and immunohistochemistry.

**Table 3:** Clinical data and ploidy assessment

Case	Histo-pathology	Ploidy	SSI	Age	FIGO 1988	FIGO 2010	Grade	Metastasis	CGH	ANCA	ANRA	Observation time	Died at month
D01	endometrioid	diploid	35, 0	83	1c	1b	1	no	3191	0.043	0	74	

D02	endometrioid	diploid	31, 7	59	1b	1a	1	no	3192	0.087	0	42	42

D03	endometrioid	diploid	45, 3	60	1c	1b	1	no	3193	0.130	0	94	

D04	endometrioid	diploid	16, 1	52	1b	1a	1	no	3194	0	0	92	

D05	endometrioid	diploid	6, 8	87	1c	1b	2	no	3195	0.043	0.043	15	15

D06*	endometrioid	diploid	13, 2	67	1b	1a	2	no	3196	0.043	0	109	

D07*	endometrioid	diploid	17, 6	78	3a	3a	2	yes	3197	0.174	0	4	4

D08*	endometrioid	diploid	13, 7	78	1b	1a	1	no	3198	0.043	0	104	

D09	endometrioid	diploid	17, 3	59	1b	1a	1	no	3193	0	0	94	

D10	endometrioid	diploid	12, 4	55	1b	1a	1	no	3200	0	0	94	

D11	endometrioid	diploid	14, 5	81	1b	1a	1	no	3201	0	0	86	

D12	endometrioid	diploid	46, 5	72	1b	1a	1	no	3202	0	0	75	

D13	endometrioid	diploid	35, 6	63	1c	1b	2	no	3203	0	0	26	26

D14	endometrioid	diploid	56, 4	51	1b	1a	1	no	3204	0	0	88	

D15	endometrioid	diploid	31, 0	53	1b	1a	2	no	3205	0	0	55	

D16	endometrioid	diploid	38, 4	66	1b	1a	3	no	3206	0.087	0	53	

Ae1	endometrioid	aneuploid	68, 0	82	1c	1b	1	no	3207	0.043	0	146	

Ae2	endometrioid	aneuploid	20, 4	80	1b	1a	1	no	3208	0.304	0	71	

Ae3	endometrioid	aneuploid	35, 2	69	1b	1a	1	no	3203	0.043	0	107	

Ae4	endometrioid	aneuploid	26, 9	46	1b	1a	2	no	3210	0.565	0.087	81	

Ae5	endometrioid	aneuploid	35, 5	55	2a	2	3	yes	3211	0.087	0	73	

Ae6	endometrioid	aneuploid	30, 7	66	1b	1a	2	no	3212	0	0	63	

Ae7	endometrioid	aneuploid	71, 0	79	1b	1a	2	no	3213	0.130	0.043	55	

Ae8	endometrioid	aneuploid	59, 6	60	1b	1a	3	no	3214	0.043	0	51	

Ae9	endometrioid	aneuploid	61, 7	72	1c	1b	3	yes	5521	0.087	0	42	

Au1	UPSC	aneuploid	70, 9	77	3a	3a	3	yes	3215	0.565	0.174	36	36

Au2	UPSC	aneuploid	42, 0	78	4b	4b	0	yes	3216	0.609	0	51	

Au3	UPSC	aneuploid	28, 5	66	1c	1b	3	no	3217	0.652	0.043	19	19

Au4	UPSC	aneuploid	59, 7	80	3a	3a	3	yes	3218	0.565	0	27	27

Au5	UPSC	aneuploid	48, 6	90	3	3a	3	yes	3219	0.130	0	9	9

Au6	UPSC	aneuploid	27, 5	88	3b	3b	0	yes	3220	0.478	0.13	17	17

Au7*	UPSC	aneuploid	59, 2	80	1a	1a	0	no	3359	0.348	0.13	7	7

Au8	UPSC	aneuploid	91, 3	83	3a	3a	0	yes	5522	0.087	0.043	10	10

* Arrays did not pass visual quality criteria and were thus excluded from further analysis.

Staging was assessed according to FIGO Committee on Gynecologic Oncology. Ploidy status was defined by image cytometry according to the Auer classification. Observation time is given in months. For CGH, the case number of the database is given http://www.ncbi.nlm.nih.gov/sky/skyweb.cgi. SSI, Stem line scatter index.

### Image Cytometry

Image cytometry was performed on Feulgen-stained paraffin-embedded histopathological slides. The staining procedure, internal standardization, and tumor cell selection were based on methods described previously [4]. All DNA-values were expressed in relation to the corresponding staining controls which were given the value 2c, denoting the normal diploid DNA-content. The tumors were classified as belonging to three groups: (i) diploid cases with a distinct peak in the normal 2c region and no cells exceeding 5c, (ii) aneuploid cases with a main peak different from 2c and a *stemline scatter index *(SSI) below or equal 8.8, and (iii) aneuploid samples with a varying numbers of cells (> 5%) exceeding 5c (SSI above 8.8). This novel classification system adheres to the parameters established by Kronenwett and colleagues, who defined the *stemline scatter index *(SSI) as a measurement of clonal heterogeneity in the tumor cell population [16].

The degree of genomic instability status in the three groups (EnD, EnA, UPSC-A) was compared with metric parameters (stemline, SSI, ANCA, ANRA) using ANOVA test. The threshold of significance was set to p < 0.05.

### Comparative Genomic Hybridization (CGH)

DNA was extracted from fresh frozen tissue using TRIzol. CGH was performed as described in detail http://www.riedlab.nci.nih.gov. Fluorescence intensity ratio plots were generated using Leica CW4000 Karyo V1.0 software (Leica Imaging Systems, Cambridge, UK). Interpretation of changes at 1pter, 16, 19, and 22 required careful examination because these loci are prone to artifacts due to the high proportion of repetitive sequences. CGH profiles of individual cases as well as the summary display of all cases can be found at http://www.ncbi.nlm.nih.gov/sky/skyweb.cgi.

### Microarray analysis

Total RNA was extracted using TRIzol (Invitrogen) followed by Qiagen RNeasy column purification (Qiagen, Valencia, CA). All samples were hybridized against the universal human reference RNA (Stratagene, La Jolla, CA, USA) using a slightly modified protocol from Hedge and colleagues [43]. Extraction and hybridization protocols used can be viewed in detail at http://www.riedlab.nci.nih.gov.

In brief, 20 μg of total RNA was reverse transcribed using random primers and converted into cDNA using reverse transcriptase. After incorporation of aminoallyl-conjugated nucleotides, the RNA was indirectly labelled with Cy3 (tumor RNA) and Cy5 (reference RNA, Amersham, Piscataway, NJ). Each sample was hybridized against the reference RNA in a humid chamber (Arraylt™ Hybridization Cassette, TeleChem Intl., Sunnyvale, CA, USA) for 16 hours at 42°C, washed, and scanned by the Axon GenePix 4000B Scanner (Axon Instruments, Union City, CA, USA). In order to account for potential amplification bias, total RNA was hybridized following the same protocol for 11 samples (20 μg each). We used customized arrays obtained from the National Cancer Institute's microarray core facility. Arrays were used from one print batch and composed of 9, 128 cDNAs denatured and immobilized on a poly-L-lysine-coated glass surface. The gene annotation file (GAL file) used (Hs-UniGEM2-v2px-32Bx18Cx18R.gal) can be found at the facility's website http://nciarray.nci.nih.gov. GenePix software version 4.0.1.17 was used to apply the GAL file through an interactive gridding process. All images of the scanned microarray slides were meticulously inspected for artifacts. Empty spots and aberrant spots and slide regions were flagged for exclusion from analyses [44].

### Microarray quality assessment and data analysis (two-group class comparison)

After discarding arrays that did not pass our visual quality filtering, a total of 13 EnD, 9 EnA, and 7 UPSC-A malignancies could be processed for further analysis. All values that did not meet the quality control criteria were treated as missing values as described in supplemental materials (Additional file 5). Intensity ratios were calculated using the background corrected median intensities that were normalized with the locally weighted scatter plot smoother (LOWESS) algorithm for each print-tip group. The fraction of data points used in the local regression (*f*) was 0.2 and other parameters were adjusted as suggested by Cleveland [45]. The value of *f *was determined using self versus self experiment. All within-slide normalized ratios were log-transformed (natural base). A total of 4, 995 genes were identified that did not show any missing values across all samples. Out of those 4, 995 genes, differentially expressed genes were identified with pair-wise analysis. To identify differentially expressed genes we used Wilcoxon rank-sum test with a permutation test (p < 0.05) and a step-wise gene selection procedure [17,46]. Genes that were identified by both approaches were selected for further analysis. Further details of applied algorithms can be found in Additional file 6.

### Biological pathway analysis

We used IPA software (v8.7, Ingenuity, Mountain View, CA) to assess the involvement of significantly differentially expressed genes and proteins in known pathways and networks. IPA determined groups of genes that together constitute networks. Such networks indicate how the genes and/or proteins of interest may influence each other above and beyond canonical pathways that are described in the Kyoto Encyclopedia of Genes and Genomes (KEGG, www.genome.jp/kegg). The IPA generated networks are listed in a certain order, with the top networks having a lower likelihood that the generation of the networks was serendipitous.

## List of abbreviations

ANCA: Average number of copy number alterations; ANRA: Average number of regional amplifications; CGH: Comparative genomic hybridization; DEG: Differentially expressed gene; DEP: Differentially expressed protein; ECM: Extracellular matrix; EnA: Aneuploid endometrioid; EnCa: Endometrial cancer; FIGO: Fédération Internationale de Gynécologie et d'Obstétrique; IPA: Ingenuity pathways analysis; KEGG: Kyoto encyclopedia of genes and genomes; PCA: Principal component analysis; SSI: Stem line scatter index; UPSC: Uterine papillary serous cancer; UPSC-A: Aneuploid uterine papillary serous cancer.

## Competing interests

The authors declare that they have no competing interests.

## Authors' contributions

JKH designed the study, performed genomic and transcriptomic experiments, and wrote the manuscript. NB performed genomic and transcriptomic experiments, analyzed results, and drafted the manuscript. TG performed result analysis and proteomics experiments, and wrote the manuscript. SH evaluated statistical data. CL drafted the manuscript and collected clinical data. DW contributed to genomics and transcripomic experiments. JD performed ploidy experiments. HPB designed the study and drafted the manuscript. BN contributed to the sample collection and ploidy experiments. UJR designed the study and drafted the manuscript. HJ helped to design the study, was involved in mass spectrometric experiments and contributed substantially to the manuscript. GA helped to design the study and drafted the manuscript. TR designed the study and wrote the manuscript. All authors read and approved the final manuscript.

## Supplementary Material

Additional file 1 **CGH data details**. Detailed list of all chromosomal imbalances of all endometrial tumors. Click here for file

Additional file 2 **Unique DEG list**. List of differentially expressed genes that were unique for all pair-wise group comparisons. Click here for file

Additional file 3 **Differential expressed protein lists**. List of all significant proteins of all pair-wise group comparisons. Click here for file

Additional file 4 **Involved genes in pathway analysis**. Presentation of all genes that are involved in identified pathways. Click here for file

Additional file 5 **Quality assessment criteria**. Quality control criteria for microarray values that had to be fulfilled for further analysis. Click here for file

Additional file 6 **Microarray data analysis (two-group class comparison)**. Details of applied algorithms for microarray data analysis. Click here for file
