# Supplementary material for: Genomic instability influences the transcriptome and proteome in endometrial cancer subtypes
Source: Mol Cancer. 2011 Oct 31;10:132. doi: 10.1186/1476-4598-10-132 (PMC3261822; doi:10.1186/1476-4598-10-132)
Supplement: Additional file 1 — CGH data details. Detailed list of all chromosomal imbalances of all endometrial tumors. [file 1476-4598-10-132-S1.DOC]

**Additional file 1: CGH data details**

| **Chrom.** | **EnD** | **EnA** | **UPSC-A** | **Gain total** | **Loss total** | **Total** |
| --- | --- | --- | --- | --- | --- | --- |
|  | n=18 | n=9 | n=8 | n=35 |  |  |
| 1p |  |  |  |  |  |  |
| 1q | gain: 6/18 33.3% | gain: 2/9 22.2% | gain: 2/8 25% | 10/35 28.5% |  | 10/35 28.5% |
| 2p |  |  | gain: 3/8 37.5% | 3/35 8.6% |  | 3/35 8.6% |
| 2q |  |  | gain: 5/8 62.5% | 5/35 14.3% |  | 5/35 14.3% |
| 3p |  |  | gain: 1/8 12.5%; loss: 2/8 25% | 1/35 2.9% | 2/35 5.7% | 3/35 8.6% |
| 3q |  |  | gain: 3/8 37.5% | 3/35 8.6% |  | 3/35 8.6% |
| 4p |  |  | loss: 1/8 12.5% |  | 1/35 2.9% | 1/35 2.9% |
| 4q |  |  | gain: 1/8 12.5%; loss: 3/8 37.5% | 1/35 2.9% | 3/35 8.6% | 4/35 11.4% |
| 5p |  |  |  |  |  |  |
| 5q |  |  | gain: 1/8 12.5%; loss: 1/8 12.5% | 1/35 2.9% | 1/35 2.9% | 2/35 5.7% |
| 6p |  |  | gain: 2/8 25% | 2/35 5.7% |  | 2/35 5.7% |
| 6q |  |  | gain: 1/8 12.5%; loss: 2/8 25% | 1/35 2.9% | 2/35 5.7% | 3/35 8.6% |
| 7p |  | gain: 1/9 11.1% | gain: 2/8 25%; loss1/8 12.5% | 3/35 8.6% |  | 3/35 8.6% |
| 7q |  | gain: 1/9 11.1% | gain: 2/8 25% | 3/35 8.6% |  | 3/35 8.6% |
| 8p | gain: 1/18 5.6% |  | gain: 1/8 12.5%; loss: 3/8 37.5% | 2/35 5.7% | 3/35 8.6% | 5/35 14.3% |
| 8q | gain: 1/18 5.6% | gain: 2/9 22.2% | gain: 5/8 62.5% | 5/35 14.3% |  | 5/35 14.3% |
| 9p |  | gain: 1/9 11.1% | gain: 1/8 12.5%; loss: 2/8 25% | 2/35 5.7% | 2/35 5.7% | 4/35 11.4% |
| 9q |  | loss: 2/9 22.2% | gain: 1/8 12.5%; loss: 1/8 12.5% | 1/35 2.9% | 3/35 8.6% | 4/35 11.4% |
| 10p |  | gain: 2/9 22.2% | gain: 1/8 12.5% | 3/35 8.6% |  | 3/35 8.6% |
| 10q |  | gain: 3/9 33.3% | gain: 2/8 25%; loss1/8 12.5% | 5/35 14.3% | 1/35 2.9% | 6/35 17.1% |
| 11p |  |  | loss: 2/8 25% |  | 2/35 5.7% | 2/35 5.7% |
| 11q |  |  | loss: 2/8 25% |  | 2/35 5.7% | 2/35 5.7% |
| 12p |  |  | gain: 1/8 12.5% | 1/35 2.9% |  | 1/35 2.9% |
| 12q |  |  |  |  |  |  |
| 13p |  |  |  |  |  |  |
| 13q |  | loss: 1/9 11.1% | gain: 1/8 12.5%; loss: 1/8 12.5% | 1/35 2.9% | 2/35 5.7% | 3/35 8.6% |
| 14p |  |  |  |  |  |  |
| 14q |  |  | gain: 1/8 12.5% | 1/35 2.9% |  | 1/35 2.9% |
| 15p |  |  |  |  |  |  |
| 15q |  | loss: 1/9 11.1% | gain: 1/8 12.5%; loss: 4/8 50% | 1/35 2.9% |  | 1/35 2.9% |
| 16p | gain: 2/18 11.1% | gain: 2/9 22.2% | gain: 1/8 12.5% | 5/35 14.3% |  | 5/35 14.3% |
| 16q | loss: 1/18 5.6% | loss: 2/9 22.2% | loss: 2/8 25% |  | 5/35 14.3% | 5/35 14.3% |
| 17p | gain: 1/18 5.6% | gain: 1/9; loss: 2/9 | gain: 1/8 12.5%; loss: 3/8 37.5% | 3/35 8.6% | 3/35 8.6% | 6/35 17.1% |
| 17q | gain: 1/18 5.6% | gain: 2/9 22.2% | gain: 5/8 62.5%; loss: 1/8 12.5% | 8/35 22.9% |  | 8/35 22.9% |
| 18p |  | gain: 1/9 11.1% | gain: 3/8 37.5% | 4/35 11.4% |  | 4/35 11.4% |
| 18q | loss: 1/18 5.6% |  | gain: 1/8 12.5%; loss: 2/8 25% | 1/35 2.9% | 3/35 8.6% | 4/35 11.4% |
| 19p |  | loss: 2/9 22.2% | gain: 1/8 12.5%; loss: 2/8 25% | 1/35 2.9% | 4/35 11.4% | 5/35 14.3% |
| 19q |  | loss: 2/9 22.2% | gain: 3/8 37.5% | 3/35 8.6% | 2/35 5.7% | 5/35 14.3% |
| 20p |  |  | gain: 5/8 62.5% | 5/35 14.3% |  | 5/35 14.3% |
| 20q | gain: 1/18 5.6% | gain: 3/9 33.3% | gain: 4/8 50% | 8/35 22.9% |  | 8/35 22.9% |
| 21p |  |  |  |  |  |  |
| 21q |  |  | gain: 1/8 12.5% | 1/35 2.9% |  | 1/35 2.9% |
| 22p |  |  |  |  |  |  |
| 22q |  | loss: 2/9 22.2% |  |  | 2/35 5.7% | 2/35 5.7% |
| Xp |  | loss: 1/9 11.1% | loss: 1/8 12.5% |  | 2/35 5.7% | 2/35 5.7% |
| Xq |  | loss: 1/9 11.1% | loss: 1/8 12.5% |  | 2/35 5.7% | 2/35 5.7% |
