# Supplementary material for: Genomic instability influences the transcriptome and proteome in endometrial cancer subtypes
Source: Mol Cancer. 2011 Oct 31;10:132. doi: 10.1186/1476-4598-10-132 (PMC3261822; doi:10.1186/1476-4598-10-132)
Supplement: Additional file 2 — Unique DEG list. List of differentially expressed genes that were unique for all pair-wise group comparisons. [file 1476-4598-10-132-S2.DOC]

**Additional file 2: Unique DEG lists**

**2a: Endometrioid diploid vs. aneuploid**

| **Spot** | **Well-ID** | **Clone** | **Map** | **Gene** | **Ratios EnD vs. EnA** | **Correlation CGH** |
| --- | --- | --- | --- | --- | --- | --- |
| 7413 | 167174 | IncytePD:2418617 | NaN | NaN | 0.31784 |  |
| 9252 | 169013 | IncytePD:1854220 | 1q24 | QSCN6 | 0.33034 |  |
| 8533 | 168294 | IncytePD:1426031 | NaN | NaN | 0.38965 |  |
| 3920 | 163681 | IncytePD:1749102 | 8p12-p11 | INDO | 0.39094 |  |
| 550 | 160311 | IncytePD:2132285 | 2p11.2 | TMSB10 | 0.39303 |  |
| 2758 | 162519 | IncytePD:566886 | 21q22.3 | CSTB | 0.40257 |  |
| 4457 | 164218 | IncytePD:2171401 | 14q22.1 | ERO1L | 0.4064 |  |
| 504 | 160265 | IncytePD:1739722 | 10p15.3-p15.2 | PFKP | 0.41218 | x |
| 5132 | 164893 | IncytePD:496003 | 18q12.1 | DSC2 | 0.41219 | x |
| 7796 | 167557 | IncytePD:635178 | 6q25.3 | SOD2 | 0.41415 |  |
| 74 | 159835 | IncytePD:2070554 | 10p15-p14 | AKR1C1 | 0.41872 | x |
| 1152 | 160913 | IncytePD:1900114 | 7q11.23 | CLDN4 | 0.43895 | x |
| 2391 | 162152 | IncytePD:2131758 | 7q11.23 | CLDN4 | 0.44753 | x |
| 1992 | 161753 | IncytePD:5033671 | 10p15-p14 | AKR1C4 | 0.45036 | x |
| 5562 | 165323 | IncytePD:3139163 | 1q25.2-q25.3 | PTGS2 | 0.46463 |  |
| 7200 | 166961 | IncytePD:2174863 | 8q13 | LYN | 0.46587 | x |
| 82 | 159843 | IncytePD:3085928 | 17q21.33 | ITGA3 | 0.47301 | x |
| 2744 | 162505 | IncytePD:1962141 | 12q12-q13 | KRT7 | 0.4914 |  |
| 7389 | 167150 | IncytePD:1922875 | 18q11.2 | ABHD3 | 0.50664 | x |
| 7509 | 167270 | IncytePD:1649959 | 12q12-q13 | KRT7 | 0.5235 |  |
| 2610 | 162371 | IncytePD:560466 | NaN | NaN | 0.53333 |  |
| 5118 | 164879 | IncytePD:86390 | 11p15.1-p14 | SAA4 | 0.53614 |  |
| 269 | 160030 | IncytePD:2790678 | 6p21 | ITPR3 | 0.54984 |  |
| 3019 | 162780 | IncytePD:417827 | 17q25.3 | DDX48 | 0.55008 | x |
| 1122 | 160883 | IncytePD:1845046 | 1q21-q22 | EFNA1 | 0.55428 |  |
| 471 | 160232 | IncytePD:468887 | 8p11.22 | ADAM9 | 0.56878 |  |
| 8062 | 167823 | IncytePD:2987878 | 22q12.2 | LIF | 0.57036 |  |
| 4772 | 164533 | IncytePD:2636514 | 7q32.2 | HIG2 | 0.57328 | x |
| 5760 | 165521 | IncytePD:3199352 | 1q21-q25 | TAGLN2 | 0.58252 |  |
| 1371 | 161132 | IncytePD:2238363 | 15q26 | ISG20 | 0.5981 |  |
| 9197 | 168958 | IncytePD:3143579 | 22 | NaN | 0.61326 |  |
| 7887 | 167648 | IncytePD:2840251 | 10q24 | IFIT4 | 0.61517 | x |
| 4445 | 164206 | IncytePD:1402615 | 16p13.3 | TNFRSF12A | 0.63732 | x |
| 7552 | 167313 | IncytePD:2211625 | 14q11.2 | PSME1 | 0.63985 |  |
| 9526 | 169287 | IncytePD:2399253 | 19p13.3 | ELA2 | 0.64316 |  |
| 8914 | 168675 | IncytePD:3872557 | 11p15.5 | HBB | 0.66739 |  |
| 1657 | 161418 | IncytePD:2680410 | 1q23 | MGST3 | 0.74852 |  |
| 8795 | 168556 | IncytePD:1805270 | Xp11.23 | USP11 | 1.3318 | x |
| 1246 | 161007 | IncytePD:1334255 | 6q13-q21 | TTK | 1.425 |  |
| **Spot** | **Well-ID** | **Clone** | **Map** | **Gene** | **Ratios EnD vs. EnA** | **Correlation CGH** |
| 15 | 159776 | IncytePD:1890902 | 6q21 | REV3L | 1.4825 |  |
| 1185 | 160946 | IncytePD:2736040 | NaN | NaN | 1.5186 |  |
| 5999 | 165760 | IncytePD:348143 | 9p24.2 | XTP5 | 1.5554 |  |
| 8179 | 167940 | IncytePD:2631284 | Xq26.3 | FLJ12649 | 1.5798 | x |
| 1225 | 160986 | IncytePD:2169635 | 3p21 | SCN5A | 1.5823 |  |
| 1282 | 161043 | IncytePD:1648517 | 17 | ATPAF2 | 1.5991 |  |
| 959 | 160720 | IncytePD:1685528 | 15q15.3 | SORD | 1.6201 | x |
| 9067 | 168828 | IncytePD:1921725 | 18q21.1 | TCF4 | 1.6429 |  |
| 6835 | 166596 | IncytePD:1376090 | NaN | NaN | 1.6441 |  |
| 6694 | 166455 | IncytePD:3002566 | 12p11 | ITPR2 | 1.6531 |  |
| 8241 | 168002 | IncytePD:2378796 | 1p35.3-p34.1 | TEKT2 | 1.6668 |  |
| 968 | 160729 | IncytePD:1967983 | NaN | NaN | 1.6689 |  |
| 2552 | 162313 | IncytePD:197908 | 13q14 | TSC22 | 1.6944 | x |
| 9964 | 169725 | IncytePD:623523 | 15 | NaN | 1.7216 | x |
| 2669 | 162430 | IncytePD:1985427 | 7p22 | ETV1 | 1.7238 |  |
| 8593 | 168354 | IncytePD:1672920 | 7q22 | PCOLCE | 1.7316 |  |
| 1878 | 161639 | IncytePD:2862836 | 12q14.3 | CPSF6 | 1.7557 |  |
| 4115 | 163876 | IncytePD:1313183 | 2p22-p21 | LTBP1 | 1.7772 |  |
| 2545 | 162306 | IncytePD:5089227 | 15q12 | SNRPN | 1.7845 | x |
| 7238 | 166999 | IncytePD:2175008 | 1q43 | NID | 1.7867 | x |
| 5854 | 165615 | IncytePD:1731716 | 9p13.3 | SPAG8 | 1.7925 |  |
| 1235 | 160996 | IncytePD:1869110 | 11q14 | PRCP | 1.8058 |  |
| 9846 | 169607 | IncytePD:1987738 | Yp11.3 | RPS4Y | 1.8154 |  |
| 3174 | 162935 | IncytePD:2499488 | 13q32.1 | DZIP1 | 1.8428 | x |
| 9347 | 169108 | IncytePD:2324195 | 3 | C3orf6 | 1.844 |  |
| 6977 | 166738 | IncytePD:1976279 | 5p13 | DAB2 | 1.8889 |  |
| 867 | 160628 | IncytePD:3543015 | 11p11.2-p11.1 | SLC43A1 | 1.8896 |  |
| 5821 | 165582 | IncytePD:1682642 | 5q14-q21 | PAM | 1.9083 |  |
| 3680 | 163441 | IncytePD:1675571 | 8p11.23 | FLJ14299 | 1.9431 | x |
| 6863 | 166624 | IncytePD:1401817 | 9q13-q21 | X123 | 1.9635 | x |
| 9228 | 168989 | IncytePD:2989991 | 17p13.1 | SERPINF1 | 1.9704 | x |
| 3045 | 162806 | IncytePD:2262662 | 4p16 | LDB2 | 1.9925 |  |
| 2674 | 162435 | IncytePD:831941 | 1p13.2 | MEP50 | 2.0122 |  |
| 9763 | 169524 | IncytePD:1613615 | 14q22-q23 | BMP4 | 2.1426 |  |
| 6238 | 165999 | IncytePD:1909488 | 2q31.1 | CYBRD1 | 2.1437 |  |
| 7384 | 167145 | IncytePD:719318 | 2p21 | CYP1B1 | 2.1613 |  |
| 600 | 160361 | IncytePD:1740542 | 5p15.2 | SEMA5A | 2.1728 |  |
| 2294 | 162055 | IncytePD:4741984 | 3p23 | SATB1 | 2.2269 |  |
| 9940 | 169701 | IncytePD:1999167 | 17q12-q21.1 | IGFBP4 | 2.3449 |  |
| 5459 | 165220 | IncytePD:551403 | 3q26.2-qter | APOD | 2.5479 |  |
| 8949 | 168710 | IncytePD:15834 | 6q23 | EPB41L2 | 2.6562 |  |
| 1979 | 161740 | IncytePD:4094885 | Xp11.4 | NDP | 2.6858 | x |
| 919 | 160680 | IncytePD:3948420 | 3q26.2-qter | APOD | 2.7451 |  |
| **Spot** | **Well-ID** | **Clone** | **Map** | **Gene** | **Ratios EnD vs. EnA** | **Correlation CGH** |
| 9749 | 169510 | IncytePD:2654926 | 18q21.1 | TCF4 | 2.8639 |  |
| 2845 | 162606 | IncytePD:583146 | 11p15.1 | PRMT3 | 2.9637 |  |
| 921 | 160682 | IncytePD:4286401 | 14q24-q32 | FLRT2 | 3.0483 |  |
| 7491 | 167252 | IncytePD:1578941 | 4q34-q35 | HPGD | 3.0728 |  |
| 4173 | 163934 | IncytePD:1525881 | 12q14.3 | CPM | 3.5486 |  |
| 1423 | 161184 | IncytePD:2589487 | 19q13.33 | NAP1 | 4.0899 | x |
| 9379 | 169140 | IncytePD:588870 | 20q13.2-q13.3 | EDN3 | 4.3388 |  |
| 7046 | 166807 | IncytePD:1505977 | 4q32-q33 | GRIA2 | 6.0075 |  |

**2b: Diploid endometrioid vs. aneuploid UPSC**

| **Spot** | **Well-ID** | **Clone** | **Map** | **Gene** | **Ratios EnD vs. UPSC-A** | **Correlation CGH** |
| --- | --- | --- | --- | --- | --- | --- |
| 9947 | 169708 | IncytePD:2506867 | 4p14 | UCHL1 | 0.097803 |  |
| 880 | 160641 | IncytePD:753651 | 10q23 | RGR | 0.13329 | x |
| 2973 | 162734 | IncytePD:2622566 | 3p25 | WNT7A | 0.23921 |  |
| 2744 | 162505 | IncytePD:1962141 | 12q12-q13 | KRT7 | 0.26454 |  |
| 8239 | 168000 | IncytePD:2842835 | 17q25 | LGALS3BP | 0.29259 | x |
| 2162 | 161923 | IncytePD:4327691 | NaN | NaN | 0.33472 |  |
| 4285 | 164046 | IncytePD:2900277 | 14q32 | IFI27 | 0.33617 | x |
| 316 | 160077 | IncytePD:3016305 | 11q22.3-q23.1 | CRYAB | 0.33708 |  |
| 7509 | 167270 | IncytePD:1649959 | 12q12-q13 | KRT7 | 0.33898 | x |
| 4126 | 163887 | IncytePD:1595081 | 9q32-q33.3 | PTGS1 | 0.34934 |  |
| 7959 | 167720 | IncytePD:1435811 | 12q13.11 | SLC38A1 | 0.34944 |  |
| 5058 | 164819 | IncytePD:1376121 | 11q13.3-q14.1 | FOLR1 | 0.36771 |  |
| 2384 | 162145 | IncytePD:1472042 | 8q24.3 | LY6E | 0.37422 | x |
| 4168 | 163929 | IncytePD:1690670 | 21q22.3 | MX2 | 0.377 | x |
| 2391 | 162152 | IncytePD:2131758 | 7q11.23 | CLDN4 | 0.38401 | x |
| 1152 | 160913 | IncytePD:1900114 | 7q11.23 | CLDN4 | 0.39355 | x |
| 649 | 160410 | IncytePD:79797 | 17q21.33 | DLX4 | 0.4029 | x |
| 855 | 160616 | IncytePD:1652785 | 17q25.2 | HUMPPA | 0.41225 | x |
| 3659 | 163420 | IncytePD:1444525 | 12q13.11 | SLC38A1 | 0.41311 |  |
| 8597 | 168358 | IncytePD:2655952 | 19p13.12 | KIAA0290 | 0.44746 |  |
| 5895 | 165656 | IncytePD:1981145 | 9q34.1 | ASS | 0.45126 |  |
| 264 | 160025 | IncytePD:1901073 | 7q35 | AKR1B1 | 0.46078 | x |
| 2442 | 162203 | IncytePD:1220385 | 17q25.3 | SOCS3 | 0.4645 | x |
| 1169 | 160930 | IncytePD:2211722 | 20q11.2 | DNMT3B | 0.46981 | x |
| 1395 | 161156 | IncytePD:1701725 | 12q24.1 | OAS1 | 0.48261 |  |
| 960 | 160721 | IncytePD:1888594 | 1q32.3 | ATF3 | 0.48685 |  |
| 8724 | 168485 | IncytePD:630458 | 8q24.3 | SLC39A4 | 0.48869 | x |
| 6445 | 166206 | IncytePD:2862971 | 1p36.33 | G1P2 | 0.49863 |  |
| 7630 | 167391 | IncytePD:734376 | 16p13.3 | MSLN | 0.5035 |  |
| 9093 | 168854 | IncytePD:1985367 | 14q32.1 | LGMN | 0.50807 | x |
| 2617 | 162378 | IncytePD:1965938 | 22 | NaN | 0.51664 |  |
| 2593 | 162354 | IncytePD:5109040 | NaN | NaN | 0.51806 |  |
| 1531 | 161292 | IncytePD:2853091 | 8q21.3 | CYP7B1 | 0.52476 | x |
| 9423 | 169184 | IncytePD:485111 | 1q32-q41 | CENPF | 0.52549 |  |
| 1882 | 161643 | IncytePD:3115514 | 2q37.2 | ARL7 | 0.53209 | x |
| 1701 | 161462 | IncytePD:1656490 | 6 | NaN | 0.53501 |  |
| 9068 | 168829 | IncytePD:2926307 | NaN | NaN | 0.5374 |  |
| 7525 | 167286 | IncytePD:1923070 | 2p25 | DDEF2 | 0.54146 | x |
| 409 | 160170 | IncytePD:523 | 19p13.2 | RNASEH2A | 0.54632 |  |
| 6601 | 166362 | IncytePD:2515389 | 19q13.2 | APOE | 0.56891 | x |
| 775 | 160536 | IncytePD:3679736 | 17q12-q21 | KRT17 | 0.57144 | x |
| 7887 | 167648 | IncytePD:2840251 | 10q24 | IFIT4 | 0.57542 | x |
| 3151 | 162912 | IncytePD:2680168 | 3q13 | PVRL3 | 0.58177 | x |
| **Spot** | **Well-ID** | **Clone** | **Map** | **Gene** | **Ratios EnD vs. UPSC-A** | **Correlation CGH** |
| 9502 | 169263 | IncytePD:1674253 | 10pter-q25.3 | SPOCK2 | 0.58195 | x |
| 4206 | 163967 | IncytePD:1907232 | 8q24 | PLEC1 | 0.59036 | x |
| 6078 | 165839 | IncytePD:2135596 | 19p13.13 | DDX39 | 0.59614 |  |
| 1085 | 160846 | IncytePD:1594108 | 11q13 | SPTBN2 | 0.60562 |  |
| 1724 | 161485 | IncytePD:3935143 | 19p13.2 | DNM2 | 0.60898 |  |
| 9930 | 169691 | IncytePD:2057823 | 19q13.43 | E2-EPF | 0.60919 | x |
| 285 | 160046 | IncytePD:1879888 | 8q24.13 | MGC3067 | 0.62045 | x |
| 8335 | 168096 | IncytePD:2496305 | 2p23 | KIF3C | 0.62885 | x |
| 2508 | 162269 | IncytePD:508926 | 19q13.11-q13.12 | CKAP1 | 0.63229 | x |
| 4108 | 163869 | IncytePD:1871201 | 12q11-q13 | KRT2A | 0.63479 |  |
| 7552 | 167313 | IncytePD:2211625 | 14q11.2 | PSME1 | 0.64675 | x |
| 8692 | 168453 | IncytePD:2056642 | 4p16.3 | TACC3 | 0.64848 |  |
| 153 | 159914 | IncytePD:1567992 | 12q13 | ACVR1B | 0.64848 |  |
| 6419 | 166180 | IncytePD:1719657 | 8p22 | MTSS1 | 0.6538 |  |
| 690 | 160451 | IncytePD:2061241 | 20cen-q13.1 | AHCY | 0.66508 | x |
| 1122 | 160883 | IncytePD:1845046 | 1q21-q22 | EFNA1 | 0.69866 |  |
| 9592 | 169353 | IncytePD:1666268 | 16q22.1 | DNCLI2 | 1.4044 | x |
| 6553 | 166314 | IncytePD:1383888 | 11p13 | LMO2 | 1.4133 | x |
| 8058 | 167819 | IncytePD:2918605 | 12p12.2 | KIAA0528 | 1.4446 |  |
| 8773 | 168534 | IncytePD:437868 | 12q21.1 | HRB2 | 1.5008 |  |
| 1600 | 161361 | IncytePD:2213276 | 9q34.3 | KIAA0625 | 1.5426 |  |
| 8531 | 168292 | IncytePD:998421 | 15q21 | TCF12 | 1.5564 | x |
| 3680 | 163441 | IncytePD:1675571 | 8p11.23 | FLJ14299 | 1.5589 | x |
| 2939 | 162700 | IncytePD:2360580 | 17q21 | LUC7A | 1.5628 |  |
| 4871 | 164632 | IncytePD:1274957 | 15q21.3-q22.31 | RAB11A | 1.566 | x |
| 2530 | 162291 | IncytePD:805942 | 9q32 | EDG2 | 1.5998 |  |
| 2096 | 161857 | IncytePD:3387976 | 13q12.13 | NUPL1 | 1.6039 |  |
| 5780 | 165541 | IncytePD:238278 | 9q33 | GSN | 1.6245 |  |
| 9730 | 169491 | IncytePD:340088 | NaN | NaN | 1.6726 |  |
| 836 | 160597 | IncytePD:1961218 | 3q25.2 | P2RY1 | 1.6759 |  |
| 5625 | 165386 | IncytePD:2912987 | 3p14.3 | CACNA1D | 1.6793 | x |
| 906 | 160667 | IncytePD:1856587 | 15 | NaN | 1.705 | x |
| 9029 | 168790 | IncytePD:11556 | 14q24.3 | TMP21 | 1.7196 |  |
| 6845 | 166606 | IncytePD:1525829 | 1q42-q44 | KMO | 1.7264 | x |
| 1104 | 160865 | IncytePD:1890794 | 1q42.13 | ITPKB | 1.7295 | x |
| 752 | 160513 | IncytePD:1998155 | NaN | NaN | 1.7329 |  |
| 9868 | 169629 | IncytePD:389707 | 9p22 | MLLT3 | 1.7349 | x |
| 1956 | 161717 | IncytePD:4225421 | 17q22 | DGKE | 1.7793 |  |
| 2408 | 162169 | IncytePD:180164 | 19p13.3 | CIRBP | 1.7823 | x |
| 2128 | 161889 | IncytePD:4309944 | 13q33.3 | NaN | 1.7834 |  |
| 959 | 160720 | IncytePD:1685528 | 15q15.3 | SORD | 1.7915 | x |
| 7010 | 166771 | IncytePD:1981939 | 16p13.3 | KIAA0643 | 1.8006 | x |
| 5706 | 165467 | IncytePD:1283532 | 6q14-q15 | TPBG | 1.8038 | x |
| 1540 | 161301 | IncytePD:2634611 | 17q22-q23.2 | PRKCA | 1.8056 |  |
| 4115 | 163876 | IncytePD:1313183 | 2p22-p21 | LTBP1 | 1.8103 |  |
| **Spot** | **Well-ID** | **Clone** | **Map** | **Gene** | **Ratios EnD vs. UPSC-A** | **Correlation CGH** |
| 6784 | 166545 | IncytePD:1403932 | 5q34-q35 | MSX2 | 1.8292 |  |
| 2674 | 162435 | IncytePD:831941 | 1p13.2 | MEP50 | 1.8393 |  |
| 3000 | 162761 | IncytePD:815861 | 6p21.2 | CDKN1A | 1.8394 |  |
| 908 | 160669 | IncytePD:1864819 | Xp22.22 | RBBP7 | 1.8648 | x |
| 4565 | 164326 | IncytePD:2888464 | 1q21.2 | TXNIP | 1.9465 | x |
| 9850 | 169611 | IncytePD:1841989 | 4q24 | PAPSS1 | 2.0115 | x |
| 6977 | 166738 | IncytePD:1976279 | 5p13 | DAB2 | 2.0472 |  |
| 8710 | 168471 | IncytePD:3254666 | 2q32.3 | HIBCH | 2.0781 |  |
| 600 | 160361 | IncytePD:1740542 | 5p15.2 | SEMA5A | 2.0842 |  |
| 7238 | 166999 | IncytePD:2175008 | 1q43 | NID | 2.1095 | x |
| 6863 | 166624 | IncytePD:1401817 | 9q13-q21 | X123 | 2.1425 |  |
| 867 | 160628 | IncytePD:3543015 | 11p11.2-p11.1 | SLC43A1 | 2.1469 | x |
| 1979 | 161740 | IncytePD:4094885 | Xp11.4 | NDP | 2.189 | x |
| 9749 | 169510 | IncytePD:2654926 | 18q21.1 | TCF4 | 2.221 |  |
| 2107 | 161868 | IncytePD:4401302 | NaN | NaN | 2.2382 |  |
| 5734 | 165495 | IncytePD:2498815 | 2q31 | GAD1 | 2.2563 |  |
| 440 | 160201 | IncytePD:698665 | 5q23.3 | SLC12A2 | 2.2676 |  |
| 9940 | 169701 | IncytePD:1999167 | 17q12-q21.1 | IGFBP4 | 2.2844 |  |
| 6505 | 166266 | IncytePD:4410949 | 4q12-q13 | CXCL5 | 2.3334 | x |
| 8949 | 168710 | IncytePD:15834 | 6q23 | EPB41L2 | 2.4203 | x |
| 9220 | 168981 | IncytePD:1636639 | 11p11.2 | CREB3L1 | 2.6426 | x |
| 4067 | 163828 | IncytePD:1737280 | NaN | NaN | 3.2749 |  |
| 9379 | 169140 | IncytePD:588870 | 20q13.2-q13.3 | EDN3 | 3.3166 |  |
| 1699 | 161460 | IncytePD:3214293 | 7p14 | ADCYAP1R1 | 3.3822 |  |
| 1554 | 161315 | IncytePD:2595612 | 11p15 | MUC5AC | 3.5522 | x |
| 4173 | 163934 | IncytePD:1525881 | 12q14.3 | CPM | 3.5529 |  |
| 5610 | 165371 | IncytePD:2242817 | 21q22.3 | TFF3 | 3.6206 |  |
| 7046 | 166807 | IncytePD:1505977 | 4q32-q33 | GRIA2 | 4.8722 | x |
| 678 | 160439 | IncytePD:685870 | 11q22-q23 | PGR | 12.2538 | x |

**2c: Aneuploid endometrioid vs. UPSC**

| **Spot** | **WellID** | **Clone** | **Map** | **Gene** | **Ratios_Ae_vs_Au** | **Correlation CGH** |
| --- | --- | --- | --- | --- | --- | --- |
| 9947 | 169708 | IncytePD:2506867 | 4p14 | UCHL1 | 0.13753 |  |
| 9084 | 168845 | IncytePD:1968446 | NaN | NaN | 0.2665 |  |
| 5058 | 164819 | IncytePD:1376121 | 11q13.3-q14.1 | FOLR1 | 0.28208 |  |
| 4126 | 163887 | IncytePD:1595081 | 9q32-q33.3 | PTGS1 | 0.31468 | x |
| 264 | 160025 | IncytePD:1901073 | 7q35 | AKR1B1 | 0.37467 | x |
| 7852 | 167613 | IncytePD:3078931 | 4q21.3 | PTPN13 | 0.42708 |  |
| 4117 | 163878 | IncytePD:1723401 | 9q32-q33.3 | PTGS1 | 0.43031 | x |
| 3174 | 162935 | IncytePD:2499488 | 13q32.1 | DZIP1 | 0.45304 | x |
| 164 | 159925 | IncytePD:2183703 | NaN | NaN | 0.4822 |  |
| 7875 | 167636 | IncytePD:2805762 | 14q11.1-q12 | SALL2 | 0.49124 | x |
| 611 | 160372 | IncytePD:460491 | 13q33 | EFNB2 | 0.51122 | x |
| 1832 | 161593 | IncytePD:2968067 | 4q32.3 | SPOCK3 | 0.51859 |  |
| 2545 | 162306 | IncytePD:5089227 | 15q12 | SNRPN | 0.52956 |  |
| 2384 | 162145 | IncytePD:1472042 | 8q24.3 | LY6E | 0.53386 | x |
| 9093 | 168854 | IncytePD:1985367 | 14q32.1 | LGMN | 0.54582 | x |
| 4331 | 164092 | IncytePD:2479074 | 11q13 | TM7SF2 | 0.55116 |  |
| 907 | 160668 | IncytePD:1910461 | 20q11.23 | CPNE1 | 0.55522 | x |
| 690 | 160451 | IncytePD:2061241 | 20cen-q13.1 | AHCY | 0.56959 | x |
| 6009 | 165770 | IncytePD:1416354 | 16q21-q22.1 | NDRG4 | 0.5715 |  |
| 8795 | 168556 | IncytePD:1805270 | Xp11.23 | USP11 | 0.60002 |  |
| 1531 | 161292 | IncytePD:2853091 | 8q21.3 | CYP7B1 | 0.60939 | x |
| 9966 | 169727 | IncytePD:1001890 | 20q11.21 | PLAGL2 | 0.61877 | x |
| 5994 | 165755 | IncytePD:2190607 | 8q24.3 | EEF1D | 0.62906 | x |
| 1808 | 161569 | IncytePD:2773217 | 17q12-q21 | MPP3 | 0.63215 | x |
| 1844 | 161605 | IncytePD:3068935 | NaN | NaN | 0.63709 |  |
| 3523 | 163284 | IncytePD:1405820 | 3 | NaN | 0.63718 | x |
| 7489 | 167250 | IncytePD:1599532 | 20q11.2-q12 | TGIF2 | 0.66356 | x |
| 3659 | 163420 | IncytePD:1444525 | 12q13.11 | SLC38A1 | 0.66807 |  |
| 2617 | 162378 | IncytePD:1965938 | 22 | NaN | 0.68093 |  |
| 8122 | 167883 | IncytePD:1798585 | 17q24-q25 | ACOX1 | 0.68567 | x |
| 8804 | 168565 | IncytePD:3186307 | 19 | NaN | 0.69092 | x |
| 8162 | 167923 | IncytePD:1688468 | 12q13 | ARF3 | 0.75279 |  |
| 6027 | 165788 | IncytePD:605831 | 12p11.23 | KCNJ8 | 1.4061 |  |
| 2096 | 161857 | IncytePD:3387976 | 13q12.13 | NUPL1 | 1.4595 |  |
| 2013 | 161774 | IncytePD:4626895 | 15q25.3-q26 | CIB1 | 1.4642 | x |
| 8741 | 168502 | IncytePD:1810777 | 11q22 | BIRC2 | 1.4752 | x |
| 4871 | 164632 | IncytePD:1274957 | 15q21.3-q22.31 | RAB11A | 1.5008 | x |
| 3000 | 162761 | IncytePD:815861 | 6p21.2 | CDKN1A | 1.5513 |  |
| 2282 | 162043 | IncytePD:1869139 | NaN | NaN | 1.563 |  |
| 964 | 160725 | IncytePD:1756503 | 1q24 | HHL | 1.59 |  |
| 9206 | 168967 | IncytePD:1751692 | 6q23.3 | KIAA1244 | 1.6348 | x |
| 7038 | 166799 | IncytePD:1814912 | 6q22.33 | MAP3K5 | 1.6483 | x |
| 2583 | 162344 | IncytePD:2131914 | 2q12-q14 | FHL2 | 1.6507 |  |
| **Spot** | **WellID** | **Clone** | **Map** | **Gene** | **Ratios_Ae_vs_Au** | **Correlation CGH** |
| 8704 | 168465 | IncytePD:1964902 | 5q33.2 | GALNT10 | 1.6625 |  |
| 8235 | 167996 | IncytePD:2633207 | 2p21-p16 | EPAS1 | 1.6809 |  |
| 7389 | 167150 | IncytePD:1922875 | 18q11.2 | ABHD3 | 1.6946 | x |
| 9515 | 169276 | IncytePD:435572 | 1q42.1-q42.2 | CHS1 | 1.7931 |  |
| 7921 | 167682 | IncytePD:1731972 | 17p11.1-q11.2 | CPD | 1.8377 |  |
| 9252 | 169013 | IncytePD:1854220 | 1q24 | QSCN6 | 1.8934 |  |
| 9485 | 169246 | IncytePD:377842 | 10q26.12 | LOC118987 | 1.9608 | x |
| 2758 | 162519 | IncytePD:566886 | 21q22.3 | CSTB | 1.9743 |  |
| 2128 | 161889 | IncytePD:4309944 | 13q33.3 | NaN | 1.9776 |  |
| 5734 | 165495 | IncytePD:2498815 | 2q31 | GAD1 | 1.9944 |  |
| 7310 | 167071 | IncytePD:1534482 | 4p12 | TXK | 2.0161 | x |
| 440 | 160201 | IncytePD:698665 | 5q23.3 | SLC12A2 | 2.121 |  |
| 5562 | 165323 | IncytePD:3139163 | 1q25.2-q25.3 | PTGS2 | 2.1722 |  |
| 5877 | 165638 | IncytePD:1513214 | 11q22 | BIRC3 | 2.3173 | x |
| 2246 | 162007 | IncytePD:4714232 | 8q11-q12 | CA8 | 2.3822 |  |
| 5118 | 164879 | IncytePD:86390 | 11p15.1-p14 | SAA4 | 2.4021 | x |
| 4820 | 164581 | IncytePD:2506090 | 8q13.3 | RDH10 | 2.6042 |  |
| 9220 | 168981 | IncytePD:1636639 | 11p11.2 | CREB3L1 | 2.6773 | x |
| 2610 | 162371 | IncytePD:560466 | NaN | NaN | 3.2671 |  |
| 7413 | 167174 | IncytePD:2418617 | NaN | NaN | 3.4535 |  |
| 9541 | 169302 | IncytePD:446969 | 3p25.1 | EAF1 | 3.5287 | x |
| 107 | 159868 | IncytePD:1861743 | 17q21.2 | HOXB13 | 3.7019 |  |
| 5610 | 165371 | IncytePD:2242817 | 21q22.3 | TFF3 | 4.0231 |  |
| 9372 | 169133 | IncytePD:2631845 | 11p15.1 | SAA1 | 5.1872 | x |
| 1554 | 161315 | IncytePD:2595612 | 11p15 | MUC5AC | 6.4478 | x |
