# Supplementary material for: Genomic instability influences the transcriptome and proteome in endometrial cancer subtypes
Source: Mol Cancer. 2011 Oct 31;10:132. doi: 10.1186/1476-4598-10-132 (PMC3261822; doi:10.1186/1476-4598-10-132)
Supplement: Additional file 3 — Differential expressed protein lists. List of all significant proteins of all pair-wise group comparisons. [file 1476-4598-10-132-S3.DOC]

**Additional file 3: Differential expressed protein lists**

**3a: Significant proteins in the comparison beteween diploid endometrioid and aneuploid endometrioid cancers**

| **SSP** | **Gene symbol** | **Protein name** | **Accession number** | **Chromosomal location** | **Correlation CGH** | **Correlation cDNA** | **Sequence coverage** | **Unique peptides** | **Expectations** | **Fold change (logarithmiert)** |
| --- | --- | --- | --- | --- | --- | --- | --- | --- | --- | --- |
|
|  |  |  |  |  |  |  |  |  |  |  |
| **Lower expression in aneuploid samples** | | |  |  |  |  |  |  |  |  |
| 1501 | VIM | Vimentin | AAA61279 | 10p13 |  |  | 68 | 39/96 | 2.8x10^-5^ | 0.77 |
| 7815 | LMNA | Laminin-A/C isoform 1 | NP_733821 | 1q22 | x |  | 31 | 16/39 | 3.8x10^-7^ | 0.49 |
| 8620 | PDIA3 | Protein disulfide-isomerase family A member 3 | NP_005304 | 15q15 | x |  | 27 | 14/42 | 7.6x10^-5^ | 0.70 |
|  |  |  |  |  |  |  |  |  |  |  |
| **Higher expression in aneuploid samples** | | | |  |  |  |  |  |  |  |
| 615 | KRT10 | Keratin 10 | AAH34697 | 17q21 | x |  | 33 | 20/80 | 5.1x10^-7^ | 1.10 |
| 1210 | KRT9 | Cytokeratin 9 | NP_000217 | 17q21 | x |  | 24 | 8/47 | 9.2x10^-3^ | 1.37 |
| 1313 | ACTB | ACTB | AAH12854 | 7p22 | x |  | 28 | 7/33 | 5.5x10^-3^ | 1.63 |
| 2006 | THEMIS | Thymocyte selection associated | CAI13396 | 6q22 |  |  | 14 | 7/74 | 0.32 | 2.68 |
| 2007 | ACTB | ACTB | AAH12854 | 7p22 | x |  | 28 | 7/33 | 5.5x10^-3^ | 1.82 |
| 2120 | ACTB | ACTB | AAH12854 | 7p22 | x |  | 28 | 7/33 | 5.5x10^-3^ | 1.59 |
| 2623 | ATP5B | ATP-Synthase | NP_001677 | 12q13 |  |  | 15 | 5/43 | 0.33 | 1.01 |
| 3230 | ACTB | ACTB | AAH12854 | 7p22 | x |  | 28 | 7/33 | 5.5x10^-3^ | 1.12 |
| 3314 | ATP5B | ATP-Synthase | NP_001677 | 12q13 |  |  | 15 | 5/43 | 0.33 | 1.16 |
| 3505 | ATP5B | ATP-Synthase | NP_001677 | 12q13 |  |  | 15 | 5/43 | 0.33 | 1.07 |
| 3806 | HSPA5 | HSP70 protein 5 | NP_005338 | 11 |  |  | 26 | 14/100 | 1.6x10^-3^ | 1.14 |
| 4006 | ACTG1 | ACTG1 protein | AAH10417 | 17q25 | x |  | 31 | 4/46 | 0.21 | 1.52 |
| 4107 | HSPB1 | HSP27 | NP_001531 | 11q22 |  |  | 33 | 7/49 | 0.037 | 1.06 |
| 4419 | ALB | Albumin | NP_000468 | 4q13 |  |  | 19 | 12/48 | 2.8x10^-5^ | 1.20 |
| 5104 | ACTB | ACTB | AAH12854 | 7p22 | x |  | 28 | 7/33 | 5.5x10^-3^ | 1.37 |
| 5230 | ANXA2 | Annexin A2 | AAH09564 | 15q22 |  | x | 43 | 15/53 | 2.9x10^-4^ | 1.92 |
| 7123 | GRB2 | Growth factor receptor-bound protein 2 isoform 1 | NP_002077 | 17q24 | x |  | 21 | 6/45 | 0.021 | 1.03 |

**3b: Significant proteins in the comparison beteween diploid endometrioid cancers and aneuploid UPSC**

| **SSP** | **Gene symbol** | **Protein name** | **Accession number** | **Chromosomal location** | **Correlation CGH** | **Correlation cDNA** | **Sequence coverage** | **Unique peptides** | **Fold change (logarithmiert)** |
| --- | --- | --- | --- | --- | --- | --- | --- | --- | --- |
|
|  |  |  |  |  |  |  |  |  |  |
| **Lower expression in aneuploid samples** | | | |  |  |  |  |  |  |
| 1607 | P4HB | Prolyl 4-hydroxylase, beta polypeptide | NP_000909 | 19q22 |  |  | 26 | 11/46 | 0.97 |
| 2302 | ANXA5 | Annexin A5 | NP_001145 | 4q27 | x |  | 56 | 19/96 | 0.84 |
| 3009 | RAB18 | RAB18 | NP_067075 | 10p12 |  |  | 30 | 6/68 | 0.96 |
| 5703 | HNRNPK | Heterogeneous nuclear ribonucleoprotein K isoform a | NP_112553 | 9q21 |  |  | 36 | 14/57 | 0.86 |
| 9301 | AKR7A2 | Aflatoxin B1 aldehyd reductase member 2 | NP_003680 | 1p36 |  | x | 32 | 11/37 | 0.96 |
|  |  |  |  |  |  |  |  |  |  |
| **Higher expression in aneuploid samples** | | | |  |  |  |  |  |  |
| 2003 | ANXA5 | Annexin A5 | NP_001145 | 4q27 |  |  | 21 | 8/48 | 1.36 |
| 2007 | ACTB | ACTB | AAH12854 | 7p22 | x |  | 28 | 7/33 | 1.82 |
| 2111 | HSP90AB1 | HSP90 alpha (cytosolic), class B | NP_031381 | 6p12 | x |  | 28 | 17/57 | 1.42 |
| 3230 | ACTB | ACTB | AAH12854 | 7p22 | x |  | 28 | 7/33 | 1.12 |
| 4006 | ACTG1 | ACTG1 protein | AAH10417 | 17q25 | x |  | 31 | 4/46 | 1.52 |
| 4315 | PDIA6 | Prot disulfide-isomerase A6 | NP_005733 | 2p25 | x |  | 31 | 9/52 | 1.53 |
| 4419 | ALB | Albumin | NP_000468 | 4q13 |  |  | 19 | 12/48 | 1.20 |
| 5104 | ACTB | ACTB | AAH12854 | 7p22 | x |  | 28 | 7/33 | 1.37 |
| 5107 | PRDX4 | Peroxiredoxin-4 | NP_006397 | Xp22 |  |  | 31 | 5/45 | 1.16 |
| 5230 | ANXA2 | Annexin A2 | AAH09564 | 15q22 |  |  | 43 | 15/53 | 1.92 |
