# Supplementary material for: Genomic instability influences the transcriptome and proteome in endometrial cancer subtypes
Source: Mol Cancer. 2011 Oct 31;10:132. doi: 10.1186/1476-4598-10-132 (PMC3261822; doi:10.1186/1476-4598-10-132)
Supplement: Additional file 4 — Involved genes in pathway analysis. Presentation of all genes that are involved in identified pathways. [file 1476-4598-10-132-S4.DOC]

**Additional file 4: Involved genes in pathway analysis**

For the comparison of diploid endometrioid- versus aneuploid endometrioid-carcinomas, 45 (83%) of the 54 DEGs were recognized in the IPA database and resulted in three networks. The highest ranked network with a score of 48 comprised 20 of the DEGs. Highlighted in bold are those presenting an **upregulated** gene: *ADAM9, AKR1C4,* ***BMP4****,* ***CYP1B1****, EIF4A3, ELANE, HBB, IDO1, ISG20, ITGA3,* ***ITPR2****, ITPR3, LIF, LYN, PCOLCE,* ***SATB1****,* ***SERPINF1****, SOD2, TNFRSF12A,* and ***TTK***. These genes interacted in a network associated with *Lipid* *Metabolism*, *Small Molecule Biochemistry*, and *Vitamin and Mineral Metabolism*. NFkB, Jnk and ERK1/2 were central nodes of this network and associated with diseases and functions regarding *Cancer*, *Hematological Disease*, and *Gastrointestional Disease* (p < 0.00001 to p < 0.0161). The second highest ranked network (score of 30) comprised 14 of the DEGs (***ATPAF2****,* ***CYBRD1****, DSC2,* ***KIAA0020****,* ***LDB2****,* ***MAP7D3****, MGST3,* ***PRCP****,* ***PRMT3****,* ***REV3L****,* ***SCN5A****, TAGLN2, TMSB10*) and was associated with *Lipid Metabolism*, *Small Molecule Biochemistry,* and *Genetic Disorder*. The third network (score of 22) comprised 11 of the DEGs (*AKR1C1****, APOD, CCDC50, CPSF6, FLRT2, HPGD, NAPSA, PAM, SORD, SPAG8, TSC22D1***) and was associated with *Gene Expression, Nutritional Disease* and *Cellular Development*.

The comparison between the *aneuploid* carcinomas being *endometrioid* or *UPSC* allowed 33 (84.6%) of all 39 DEGs for IPA analysis. Three overlapping networks reached the level of significance with a score of 28 to 22. The top network (Score of 28, *ACOX1, ARF3,* ***BIRC2****,* ***CPD****,* ***EAF1****, EEF1D, MPP3, NDRG4, RABGAP1L,* ***SAA1****, SPOCK3,* ***TXK***) was associated with *Cardiovascular System Development and Function, Cell Cycle, Lipid Metabolism* and particularly with the canonical pathways *Gene Expression* (p < 0.0245) and *Cell Death* (p < 0.0338). This network interacts via ***BIRC2****, SAA*, and ***SAA1*** with network two (Score of 28 and associated with *Cell Death*, *Cellular Movement*, and *Hematological System Development and Function;* ***BIRC2****,* ***BIRC3****, CPNE1, EFNB2,* ***EPAS1****,* ***FHL2****,* ***KCNJ8****,* ***MAP3K5****, PTPN13,* ***SAA1****, SALL2, TM7SF2*) and via *CTSH, ITPR2*, and ***SAA1*** with network 3 (Score of 22 and associated with *Cellular Assembly and Organization*, *Cellular Function and Maintenance*, and *Cell Signalling;* ***CA8****,* ***CIB1****,* ***CSTB****,* ***GALNT10****,* ***HOXB13****,* ***LYST****, PLAGL2,* ***RDH10****,* ***SAA1****, TGIF2*)(**Figure 3b**). Interestingly, ***SAA1*** connected as well network 2 and network 3 with each other. IFNG, TGFB, MYC, and NFkB act as central nodes in these networks.

In comparisons of endometrioid diploid *versus* the UPSC aneuploid – a total of 67 (88.2%) of the 76 DEGs were part of the IPA database. Here, we could define five overlapping networks with the first one reaching a score of 44 including 20 of the DEGs (***ADCYAP1R1,*** *APOE****,*** *ATF3****,*** *CRYAB****, CXCL5,*** *DNM2****, DYNC1LI2, GSN,*** *IFI27****,*** *ISG15****, ITPKB,*** *KRT17****, LPAR1,*** *MTSS1****,*** *MX2****,*** *OAS1****, PRKCA,*** *RNASEH2A****,*** *SOCS3****, TXNIP***) being associated with pathways of *Organismal Injury and Abnormalities*, *Cardiac Necrosis/Cell Death*, and *Cell Death*. Network 2 obtained a score of 28, comprised 14 DEGs (*ACVR1B,* ***CLUAP1****, CYP7B1, DERL1, DLX4, FCHO1,* ***GSN****,* ***HIBCH****, KIF3C,* ***KMO****, MSLN, PLEC,* ***SETX****, SLC39A4*) and revealed *Organ Morphology*, *Reproductive System Development and Function*, and *Skeletal and Muscular Disorders* pathways. The third network reached a score of 20, comprised 11 of the DEGs (*CENPF, DDX39,* ***DGKE****, DNMT3B,* ***KRR1****,* ***LUC7L3****,* ***MSX2****, SLC38A1,* ***TCF12****, UBE2S, WNT7A*) and was associated with *Cellular Development, Cellular Growth and Proliferation,* and *Cancer*. The fourth highest ranked network reached a score of 19, comprised 11 of the DEGs (*ARL4C****,*** *ASS1****, CACNA1D,*** *KRT2****,*** *LGALS3BP****, LMO2, MLLT3, P2RY1, PAPSS1, RBBP7,*** *SPOCK2*) and was associated with *Cardiovascular Disease, Hematological Disease,* and *Skeletal and Muscular Disorders.* The fifth network consists of nine DEGs (*ASAP2,* ***CIRBP****,* ***PGR****, PVRL3, SPTBN2, TACC3, TBCB,* ***TMED10****,* ***TPBG***) and reached a score of 15. Associated network functions were *Endocrine System Development and Function*, *Small Molecule Biochemistry*, and *Gene Expression*. All networks were associated with *Cancer (p < 0.0196)*, Genetic Disorder (p < 0.0234), *Cellular Growth and Proliferation* (p < 0.0212)*,* and *Cell-To-Cell Signalling and Interaction* (p < 0.0234). Three remaining networks obtained one focus gene only and all failed the level of significance with a score < 5.
