# Supplementary material for: Genomic instability influences the transcriptome and proteome in endometrial cancer subtypes
Source: Mol Cancer. 2011 Oct 31;10:132. doi: 10.1186/1476-4598-10-132 (PMC3261822; doi:10.1186/1476-4598-10-132)
Supplement: Additional file 5 — Quality assessment criteria. Quality control criteria for microarray values that had to be fulfilled for further analysis. [file 1476-4598-10-132-S5.DOC]

**Additional file 5: Quality assessment criteria**

From GenePix result-file the following features were utilized for quality filtering.

1. Number of saturated pixels in signal (F) is greater than 20%.

2. Diameter of a spot is less than 50 pixels.

3. Spot is empty.

4. Number of pixels is less than 40.

5. Both test and reference median intensities below 100.

6. Percentage of the pixels not above background is below 40% for either channel.

If any of the conditions above fulfilled, corresponding spot was deleted.
