# Supplementary material for: Genomic instability influences the transcriptome and proteome in endometrial cancer subtypes
Source: Mol Cancer. 2011 Oct 31;10:132. doi: 10.1186/1476-4598-10-132 (PMC3261822; doi:10.1186/1476-4598-10-132)
Supplement: Additional file 6 — Microarray data analysis (two-group class comparison). Details of applied algorithms for microarray data analysis. [file 1476-4598-10-132-S6.DOC]

**Additional file 6: Microarray data analysis (two-group class comparison)**

Intensity ratios were calculated using the background corrected median intensities that were normalized with the locally weighted scatter plot smoother (LOWESS) algorithm for each print-tip group. The fraction of data points used in the local regression (f) was 0.2 and other parameters were adjusted as suggested by Cleveland [45]. The value of f was determined using self versus self experiment. All within-slide normalized ratios were log-transformed (natural base). A total of 4,995 genes were identified that did not show any missing values across all samples. Out of those 4,995 genes, differentially expressed genes were identified with pair-wise analysis: diploid endometrioid- versus aneuploid endometrioid-cancer, aneuploid endometrioid-cancer versus aneuploid UPSC and diploid endometrioid-cancer versus aneuploid UPSC. In order to produce a robust gene list, we used two methods and chose genes only when they appeared in both tests. First, we conducted the Wilcoxon rank-sum test with a permutation test so that if the p-value between the two groups was below 0.05, the values were randomly labeled into these two groups and the p-value was computed and repeated 10,000 times. All cases where the p-value using permuted labels was under 0.05 were summed and divided by the total number of permutations (10,000). This p-value denotes the probability that a gene had a smaller or equal significance by random permutation than the original significance as described earlier. Genes having p‑value below 0.05 were considered to be differentially expressed. Second, we utilized a step-wise gene selection procedure [17, 46]. The basic idea is to add genes one by one to a set of genes that discriminates two classes the best using Fisher’s linear discriminant. The step-wise procedure was stopped if the weight (that marks the separation of the ratios between two classes) was less than 0.001. Finally, we took an intersection of the genes that were statistically significant using Wilcoxon test and also identified with the step-wise gene selection procedure.
